# Knowledge domain and emerging trends in HIV-MTB co-infection from 2017 to 2022: A scientometric analysis based on VOSviewer and CiteSpace

**DOI:** 10.3389/fpubh.2023.1044426

**Published:** 2023-02-01

**Authors:** Miaona Liu, Wei Li, Wenmei Qiao, Limian Liang, Zhaoqin Wang

**Affiliations:** ^1^Department of Pharmacy, The Third People's Hospital of Shenzhen, Shenzhen, China; ^2^National Center for Infectious Disease Research, The Third People's Hospital of Shenzhen, Shenzhen, China

**Keywords:** human immunodeficiency virus (HIV), *Mycobacterium tuberculosis* (MTB), scientometric analysis, infectious disease, VOSviewer, CiteSpace

## Abstract

Co-infection with *Mycobacterium tuberculosis* (MTB) in human immunodeficiency virus (HIV)-infected individuals is one of the leading causes of death. Also, research on HIV and MTB (HIV-MTB) co-infection was found to have a downward trend. In this work, we performed the knowledge domain analysis and visualized the current research progress and emerging trends in HIV-MTB co-infection between 2017 and 2022 by using VOSviewer and CiteSpace. The relevant literatures in this article were collected in the Web of Science (WoS) database. VOSviewer and CiteSpace bibliometric software were applied to perform the analysis and visualization of scientific productivity and frontier. Among all the countries, USA was dominant in the field, followed by South Africa, and England. Among all the institutions, the University of Cape Town (South Africa) had more extensive collaborations with other research institutions. The *Int J Tuberc Lung Dis* was regarded as the foremost productive journal. Survival and mortality analysis, pathogenesis, epidemiological studies, diagnostic methods, prognosis improvement of quality of life, clinical studies and multiple infections (especially co-infection with COVID-19) resulted in the knowledge bases for HIV-MTB co-infection. The clinical research on HIV-MTB co-infection has gradually shifted from randomized controlled trials to open-label trials, while the cognition of HIV-TB has gradually shifted from cytokines to genetic polymorphisms. This scientometric study used quantitative and qualitative methods to conduct a comprehensive review of research on HIV-MTB co-infection published over the past 5 years, providing some useful references to further the study of HIV-MTB co-infection.

## 1. Introduction

Acquired human immunodeficiency syndrome (AIDS) is an infectious disease caused by the HIV. MTB is one of the common opportunistic infections in HIV patients and is an important influencing factor in disease progression. It is well-known that MTB increases levels of HIV-1 replication, transmission, and genetic diversity. Moreover, HIV-1 infection not only alters the course of MTB infection, but also significantly increases the risk of active MTB (1). In fact, previous studies have reported TB in 40% of patients who died of HIV-1 (2). Therefore, HIV-MTB co-infection provides reciprocal advantages to both pathogens (3). Currently, there is a large body of literature on this complex disease. The combing of the literature is critical to a comprehensive understanding of the disease and provides practitioners with a more in-depth perspective.

Bibliometrics is the study of academic publishing that can be used to quantify the impact of independent research findings and the development of literature in a field of study or a specific disease. Furthermore, bibliometric analysis also uses statistics to describe the tendencies of scientific research (4, 5). This type of research can summarize the status and development trends of specific disciplines by analyzing the different characteristics of the literature (country, institution, journal, author, keywords, references, etc.), and provide directions and ideas for future research (5). VOSviewer (6) and CiteSpace (7) are relatively cutting-edge bibliometric analysis software. WoS is one of the commonly used bibliometric analysis databases.

We used CiteSpace and VOSviewer software to visualize country distribution, author and co-cited authors, journals and co-cited journals, co-cited references, keyword cluster analysis, and timeline, and provides a reference for combining research frameworks and expanding new ideas and methods in this field.

## 2. Methods

### 2.1. Data source and search strategy

We searched WoS to collate HIV-MTB co-infection-related studies published between 2017 and 2022 (until March 24, 2022); the literature retrieval time was March 18, 2022. The database source was limited to the Web of Science Core Collection, and there were no restrictions on publication types, publication language, etc. “Topic” was selected as the search type, and the main search terms were as follows: “acquired immune deficiency syndrome,” “human immunodeficiency virus,” “tuberculosis,” “mycobacteria,” and “non-tuberculosis mycobacteria.” All eligible literature obtained from the WoS was downloaded, and scientometric tools were used for further analysis.

### 2.2. Statistical analysis

The data were cleaned before analysis by two professionals, following which the meaningless keywords were removed, and the keywords with the same meaning were merged (single and plural, synonym, abbreviation, etc.).

Productive countries/regions, journals, institutions, and authors were identified using VOSviewer (1.6.16), while the main co-cited journals, authors and references, and related visual networks were also constructed. Clustering analysis of high-frequency keywords based on clustering function is constructed using CiteSpace (5.6.R4).

Microsoft Office Excel 2019 (Microsoft Corporation, Redmond, WA, USA) was used to manage data and analyze publishing trends. Use the linear model *f* (x) = ax + b to predict the number of studies in 2022. The variable *f* (x) represents the number of studies and x represents the year of publication. In the VOSviewer network graph, the number of studies or the co-occurrence frequency is reflected by the size of the node, the co-occurrence relationship is represented by the link between the nodes, and the co-occurrence frequency of two nodes is represented by the size of the link. Impact factors (IFs) for academic journals were collected from the 2021 Journal Citation Reports (JCR; Clarivate Analytics, Philadelphia, PA, USA).

**Figure 1:**
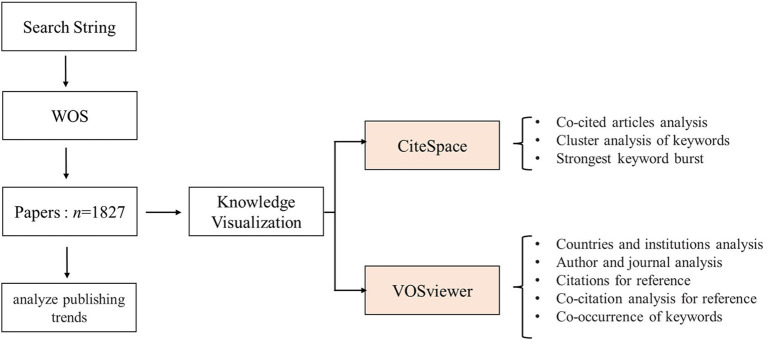
The methodological flow diagram of the study. WOS, Web of Science.

The flow chart of the study is shown in Figure 1.

## 3. Results

### 3.1. Annual growth trend of publications

**Figure 2:**
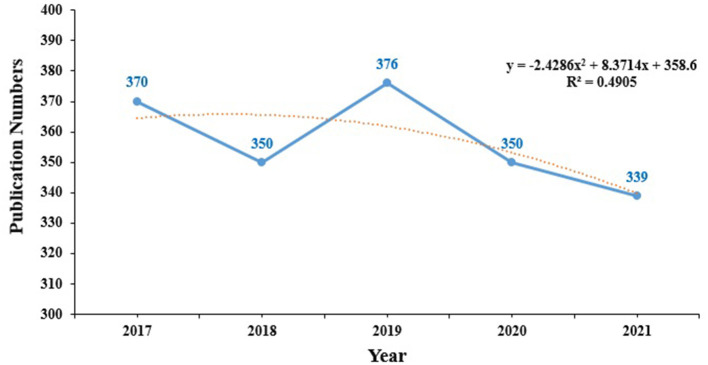
The output of articles and growth prediction of HIV-MTB co-infection research. The blue solid line represents the number of articles published between 2017 and 2021, and the yellow dotted line represents the linear fitting curve predicting the expected articles.

The WoS database shows that 1,827 articles were published between 2017 and 2022. These articles were cited as 13,897 times, with an average of 7.61 citations per article. The number of publications devoted to HIV-MTB co-infection has gradually decreased from 2017 to 2021, and the linear fitting of publications revealed a significant correlation. The model fitting curve of the growth trends of the cumulative number of published articles is shown in Figure 2 (2017–2021). Although there was a slight increase in 2019, the average annual number of publications was found to be <360 over the last 5 years.

### 3.2. Contribution of countries and institutions

A total of 1,827 publications on HIV-MTB co-infection were coauthored by 3,042 institutions from 135 countries/regions. Table 1 shows the 10 most productive countries and institutions. The USA was the leading country, with 590 published articles and 6,509 citations. Four of the top 10 institutions are from South Africa, indicating that HIV-MTB co-infection in South Africa has been extensively studied.

**Table 1:** The top 10 countries and institutes that contributed to publications about HIV-MTB co-infection.

**Rank**	**Country/region**	**Documents**	**Citations**	**Rank**	**Institution/country**	**Documents**	**Citations**
1	USA/North America	590	6,509	1	Univ Cape Town/South Africa	141	1,971
2	South Africa/Africa	376	3,934	2	Univ Witwatersrand/South Africa	92	1,213
3	England/Europe	251	3,336	3	London Sch Hyg and Trop Med/England	86	1,020
4	India/Asia	183	715	4	Stellenbosch Univ/South Africa	74	1,037
5	Ethiopia/Africa	115	739	5	Johns Hopkins Univ/USA	69	989
6	China/Asia	110	687	6	Univ KwaZulu Natal/South Africa	69	687
7	Brazil/South America	91	960	7	Makerere Univ/Uganda	48	492
8	France/Europe	88	900	8	Ctr Dis Control and Prevent/China	42	724
9	Australia/Oceania	85	877	9	UCL/England	40	854
10	Netherlands/Europe	78	809	10	Emory Univ/USA	35	601

**Figure 3:**
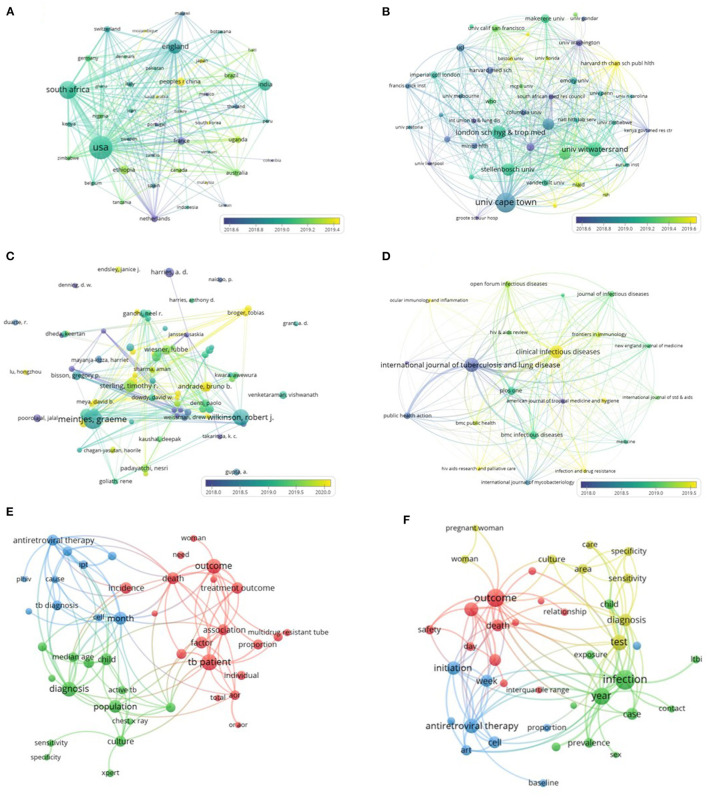
The distribution of countries, institutions, authors, and journals on HIV-MTB co-infection research. **(A)** Map of countries with publications on HIV-MTB co-infection. **(B)** Map of institutions with publications on HIV-MTB co-infection. **(C)** Map of authors with publications on HIV-MTB co-infection. **(D)** Map of journals with publications on HIV-MTB co-infection. **(E)** The keywords of articles about HIV-MTB co-infection published by *Int J Tuberc Lung Dis*. **(F)** The keywords of articles about HIV-MTB co-infection published by *Clin Infect Dis*. The nodes represent the countries, institutions, authors, and journals. The depth of color represents the publication year of the included articles. The size of the dot represents the number of publications. The thickness of the connecting lines represents the strength of collaboration in the countries, institutions, authors, and journals.

The co-occurrence network map was constructed using countries (44/135, 32.6%) and institutions (45/3,042, 1.5%) with a number of publications ≥15 (T ≥ 15; shown in Figures 3A, B). The figures showed that the USA, South Africa, England, and Brazil had larger sizes of nodes and thicker lines, which indicated that they had more publications and more collaborations. The University of Cape Town (South Africa) published the largest number of articles and had the most citations in all the journals. With reference to time, China is currently producing considerable research on HIV-MTB co-infection, as can be interpreted from different colors representing different times.

### 3.3. Authors' and journals' cooperation network

The number of articles published collaboratively by 10,426 authors is 1,827. Table 2 shows the top 10 most prolific authors and their citations for HIV-MTB coinfection. As shown in Figure 3C, the authors (80/10,426, 0.8%) with at least five publications (T ≥ 5) were further included to construct a co-occurrence network map. Graeme Meintjes is the most published author and co-author with 27 publications and 337 citations.

**Table 2:** The top 10 authors who contributed to publications about HIV-MTB co-infection.

**Documents rank**	**Citations rank**	**Author**	**Documents**	**Citations**	**Institution/country**
1	1	Meintjes	27	337	Univ Cape Town/ South Africa
2	2	Wilkinson	22	276	Univ Cape Town/ South Africa
3	19	Maartens	15	81	Univ Cape Town/ South Africa
4	4	Sterling	15	212	Stellenbosch Univ/USA
5	41	Andrade	11	47	Vanderbilt Univ/USA
6	63	Gupta	11	29	Johns Hopkins Univ/USA
7	9	Harries	11	116	London Sch Hyg and Trop Med, London/England
8	30	Wiesner	11	56	Wiesner
9	33	Bisson	10	52	Univ Penn/USA
10	10	Dooley	10	115	Johns Hopkins Univ/USA

A total of 1,827 articles were published by 669 journals. In Table 3, the top 10 most productive journals and their citations on HIV-MTB co-infection are shown. As shown in Figure 3D, a co-occurrence network map was constructed using journals with a number of publications ≥10 (T ≥ 10; 21/669, 3.1%). The *Int J Tuberc Lung Dis* (IF 2021 = 7.450, Q1) published the largest number of articles; however, the *Clin Infect Dis* had more extensive cooperating relationships with other institutions; this was also (IF 2021 = 9.079, Q1) the journal with the highest IF on HIV-MTB co-infection. The keywords of articles published in important journals *Int J Tuberc Lung Dis* and *Clin Infect Dis* were further analyzed, as shown in Figures 3E, F.

**Table 3:** The top 10 journals and co-cited journals about HIV-MTB co-infection.

**Documents rank**	**Citations rank**	**Journal**	**Documents**	**Citations**	**IF**	**Q**	**Country**
1	1	*Int J Tuberc Lung Dis*	152	1,263	2.373	Q2	France
2	3	*Clin Infect Dis*	133	1,404	9.079	Q1	USA
3	4	*BMC Infect Dis*	52	332	3.090	Q3	England
4	5	*PLoS One*	51	377	3.240	Q2	USA
5	6	*Public Health Action*	35	179	0	Not recorded	USA
6	7	*Open Forum Infect Dis*	32	155	3.835	Q3	USA
7	8	*J Infect Dis*	30	479	5.226	Q1	USA
8	9	*Front Immunol*	21	255	7.561	Q2	Switzerland
9	10	*Am J Trop Med Hyg*	20	150	2.345	Q2	USA
10	45	*Cureus*	19	20	0	Not recorded	USA

IF, impact factor; Q, Quartile in Category.

### 3.4. Reference citations, reference co-citation analysis (RCA), and representative articles

**Figure 4:**
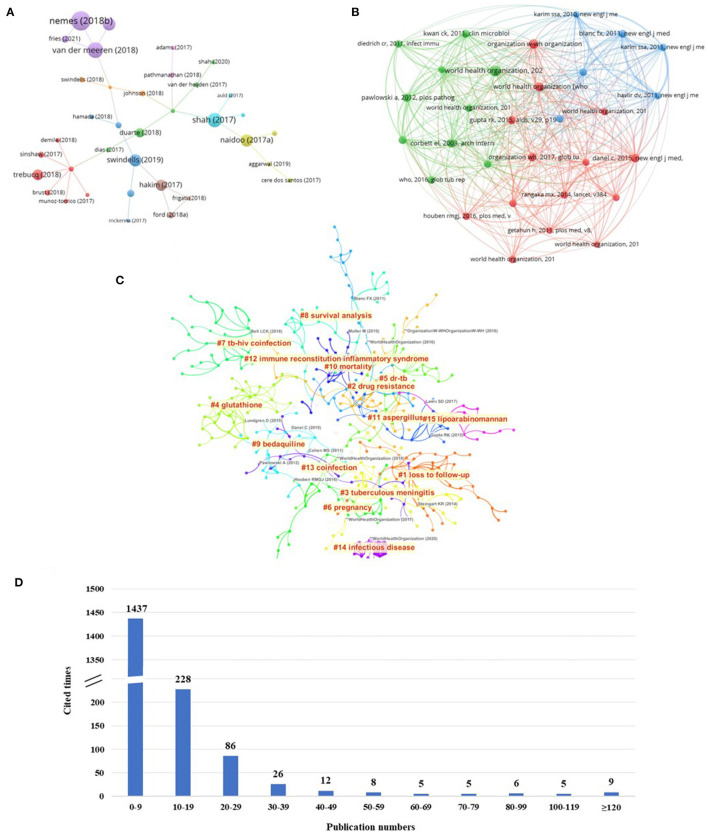
Reference citations and RCA of HIV-MTB co-infection research. **(A)** Analysis of citation times of references on HIV-MTB co-infection. **(B)** Map of the analysis of co-cited references on HIV-MTB co-infection. **(C)** Map of RCA on HIV-MTB co-infection. **(D)** The cluster map of RCA on HIV-MTB co-infection. RCA, reference co-citation analysis.

Reference citations can reflect the core articles in the research field to a certain extent. As shown in Figure 4A, 1,827 articles are analyzed by reference citations. As shown in Figure 4B, most of the articles (1,437/1,827, 45.7%) were cited 0–9 times, and the articles (390/1,827, 21.3%) with no <10 citations (T ≥ 10) were used for analysis and generated co-occurrence network map. The top 20 papers (3, 8–26) with citations ≥80 (T ≥ 80) were analyzed, as shown in Table 4.

**Table 4:** The top 20 highly cited articles about HIV-MTB co-infection.

**Rank**	**References**	**Citations**
1	Nemes et al. (8)	291
2	Marcolino et al. (9)	287
3	Van Der Meeren et al. (10)	189
4	Shah et al. (11)	156
5	Nunn et al. (12)	151
6	Lewinsohn et al. (13)	141
7	Tait et al. (14)	128
8	Kaushik and Lebwohl (15)	126
9	Swindells et al. (27)	122
10	Naidoo et al. (17)	113
11	Cassini et al. (28)	110
12	Paladini and Pollini (19)	105
13	Gause et al. (20)	104
14	Havlir et al. (21)	102
15	Hakim et al. (22)	99
16	Mayer-Barber and Yan (23)	97
17	Trébucq et al. (24)	96
18	Volz and Sutter (25)	90
19	Glaziou et al. (26)	88
20	Büttner et al. (27)	80

RCA means that the relationship between articles is expressed through frequently cited articles; the more frequently two articles are co-cited, the stronger the connection between them (27). Hence, RCA is often used to explore research hotspots and co-citation correlations among articles in a given academic field, and generalize these data to create major clusters. Figure 4C shows the knowledge map of co-cited articles. As shown in Figure 4D, the co-citation of the articles was clustered into 15 hotspots.

### 3.5. Analysis of keywords

**Figure 5:**
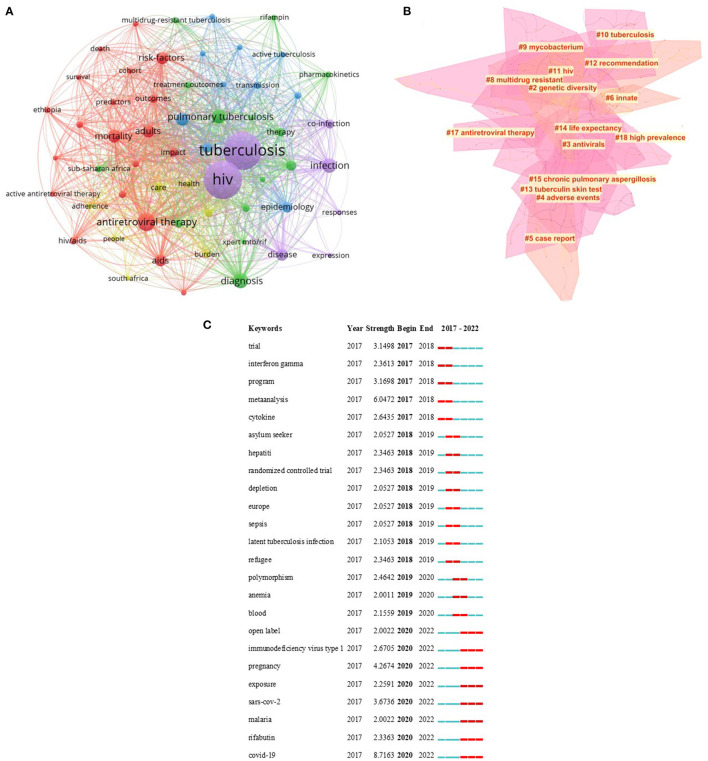
Analysis of keywords on HIV-MTB co-infection. **(A)** The co-occurrence analysis of keywords on HIV-MTB co-infection. **(B)** The cluster map of keywords on HIV-MTB co-infection. **(C)** The burst keywords of keywords on HIV-MTB co-infection. The nodes represent the keywords. The depth of color represents the topic of keywords. The size of the dot represents the number of keywords. The thickness of the connecting lines represents the strength of the correlation in the keywords.

A total of 6,418 keywords were identified in 1,827 articles. In order to better understand the connection between them, the keywords with similar meanings are combined for analysis. As shown in Figure 5A, a co-occurrence network map is constructed for keywords whose frequency is not <20 (T ≥ 20). As shown in Figure 5B, 15 clusters were obtained by clustering the keywords using the K-means algorithm. The value of *S* was 0.8015, and an *S*-value > 0.5 indicates reasonable clustering. The value of *Q* was 0.7831, and it is generally believed that *Q* > 0.3 suggests the clustering structure is significant.

The analysis was stratified by year of publication to analyze the strongest keyword bursts. As shown in Figure 5C, the strongest keyword bursts were analyzed according to the year of publication.

## 4. Discussion

According to 2017–2022 data in WOS, 1,827 articles on HIV-MTB co-infection were published in 669 journals, of which 31,723 references were cited by 3,042 institutions from 135 countries. To better understand the development of HIV-MTB coinfection over the past 5 years, the knowledge domain and emerging trends in HIV-MTB coinfection were analyzed using VOSviewer and CiteSpace through a scientometric study.

The annual output of HIV-MTB co-infection-related publications was found to show a generally decreasing trend over the past 5 years. However, nearly 321 studies are estimated to be published by 2022 based on the linear fit in Figure 2. The United States, South Africa, England and Brazil, France are the top five high-producing countries, and the United States is the leading country. Cooperation existed between countries; the United States has the strongest cooperation with South Africa, followed by South Africa with England, and the United States with England. The countries that have been actively pursuing HIV-MTB co-infection research over recent years are China, followed by South Korea, and Japan, as shown in Figure 3A.

A total of 1,827 publications on HIV-MTB co-infection were published by 3,042 institutions and 10,426 authors. A total of 9,269 (9,269/10,426, 88.9%) authors published only one paper, showing that HIV-MTB co-infection research was broadly performed but in a somewhat scattered way and not in-depth. Yet, there is a stable research team working on HIV-MTB co-infection at present. Also, Countries that have published a large number of articles have core institutions with their own representative authors. The University of Cape Town (South Africa) should be considered the core institution for HIV-MTB co-infection research, which has the highest number of articles and citations and closer associations with other institutions.

With the most publications, citations, and collaborations, Graeme Meintjes from the University of Cape Town is undoubtedly the most active author. *International Journal of Tuberculosis and Lung Disease* (IJTLD), the official journal of the International Union Against Tuberculosis and Lung Disease (IUATLD), resulted as the most productive journal; as the official publication of the *Infectious Diseases Society of America* (IDSA), *Clinical Infectious Diseases* (CID) was the journal with the highest IF in the field of HIV-MTB co-infection. We analyzed the two journals and found that both focused on disease populations, diagnosis, treatment, and outcomes. The difference between them is that the *International Journal of Tuberculosis and Lung Disease* pays more attention to tuberculosis patients, while *Clinical Infectious Diseases* pays more attention to infectious disease patients.

From the perspective of reference citations, nearly 80% of the papers were cited 0–9 times and nine papers were cited more than 120 times. Since these studies are highly authoritative and credible, they should be given enough attention. The main themes of concern were diagnosis, outcome, treatment, prevention, immunology, epidemiology, or health care sciences services. The literature on vaccines related to tuberculosis and HIV was published by Nemes et al. (8) is the core paper in the field of HIV-MTB co-infection, reflecting there is high interest and importance in developing promising candidates for a single vaccine to combat both diseases.

Researchers pay close attention to publications that have strong citation bursts over a period of time. Herein, we summarized the emerging topics about clinical (diagnosis, treatment, outcome, and prevention) epidemiology, immunology, and experimental studies of HIV infection combined with tuberculosis over the past 5 years based on RCA and keywords. Lipoarabinomannan (LAM) is a focus of interest for the diagnosis of AIDS opportunistic infection tuberculosis. LAM is an important component of the cell wall of *Mycobacterium tuberculosis*, which has important and unique immunomodulatory properties and is a tool for diagnosing latent infection of MTB and monitoring the effectiveness of anti-TB treatment (28). Tuberculous meningitis (TBM), the central nervous system tuberculosis caused by MTB infection, is the most severe complication of TB (29). TBM has a high fatality rate, mainly due to insufficient diagnostic testing (30). Although the World Health Organization recommends the GeneXpert MTB/RIF assay technology, its sensitivity is still <60% (31). LAM assays in cerebrospinal fluid and pleural effusion are also currently under investigation, while the lateral flow assay for TB lipoarabinomannan is easy to use; however, its detection in cerebrospinal fluid has low sensitivity (32). Therefore, the development of new detection techniques for the early diagnosis of TBM is of urgent importance, and the search for new biomarkers has become an important direction of current research. With the application of antiretroviral therapy, people living with HIV face a reduction in tuberculosis-related mortality; nevertheless, with the widespread introduction of highly active antiretroviral therapy, the incidence of immune reconstitution inflammatory (IRIS) has been found to rapidly grow. Its important characteristics are high incidence, unclear pathogenesis, diverse manifestations, and non-specific treatment. It has become a focus of interest in AIDS research worldwide (32, 33). In addition, while establishing co-treatment for HIV-MTB, drug interactions involving rifamycins (rifampin and rifapentine) for drug-sensitive MTB or bedaquiline for drug-resistant MTB need to be taken into account. Moreover, attention should also be paid to the treatment of drug-induced liver damage caused by anti-MTB drugs with reduced glutathione. Furthermore, postpartum women are at especially high risk of MTB disease, likely because of immunologic changes that occur late in pregnancy and allow latent MTB infection to progress to MTB disease that becomes clinically apparent after delivery (34).

By analyzing the history of AIDS and tuberculosis over the past 5 years, we found that the study of adverse events (drug-induced liver damage caused by anti-tuberculosis drugs), drug interactions, and co-infection have always been the focus of research; however, different aspects were emphasized at different stages. The clinical research also gradually changed from randomized controlled trials to open-label trials, and the cognition of AIDS-MTB has gradually shifted from cytokines to genetic polymorphisms. Since 2020, affected by the new crown epidemic, patients with triple infection of HIV-MTB and COVID-19 were found, thus presenting new challenges in HIV-MTB treatment.

At present, although a lot of mechanistic and drug research has been conducted, most drugs are still used to improve clinical symptoms. In addition, much work remains to be done to reduce the spread of new infections, improve treatment efficacy and tolerability, and identify and manage drug interactions, most notably in developing relevant vaccines.

## 5. Conclusions

This paper comprehensively used CiteSpace and VOSviewer software to analyze the knowledge base and main research interest in HIV-MTB co-infection publications between 2017 and 2022. The USA contributed the most to the HIV-MTB co-infection research, and the University of Cape Town (South Africa) and Witwatersrand University (South Africa) result as the core institutions. Prof. Meintjes is the core researcher. *Int J Tuberc Lung Dis* and *Clin Infect Dis* are the significant journals for HIV-MTB co-infection. Epidemiology and clinical research on adverse events, complications, and co-infection are recent major topics in HIV-MTB co-infection research. In addition, recent research topics about the prevention of HIV-MTB coinfection have focused on exploring microscopic mechanisms and developing new therapeutic strategies. This scientometric review provides a comprehensive overview of HIV-MTB co-infection-related research published from 2017 to 2022, which could provide valuable references for researchers. As researchers obtain more important information on HIV-MTB co-infection, the prevention, diagnosis, management, and prognosis of HIV-MTB co-infection will soon become more effective and efficient.

## Author contributions

All authors listed have made a substantial, direct, and intellectual contribution to the work and approved it for publication.
